# A direção da ciência na ditadura: um perfil dos dirigentes da Capes, do CNPq e da Finep, 1964-1985

**DOI:** 10.1590/S0104-59702024000100075

**Published:** 2025-02-10

**Authors:** Maria Caramez Carlotto, Demétrio G.C. de Toledo

**Affiliations:** i Professora, Universidade Federal do ABC. São Bernardo do Campo – SP – Brasil. maria.carlotto@ufabc.edu.br; ii Professor, Universidade Federal do ABC. São Bernardo do Campo – SP – Brasil. demetrio.toledo@ufabc.edu.br

**Keywords:** Políticas de ciência, tecnologia e inovação, Dirigentes, Gestão da ciência, tecnologia e inovação, Ditadura militar, Science, technology and innovation policies, General directors, Science, technology and innovation management, Military Dictatorship

## Abstract

Este artigo apresenta uma prosopografia dos dirigentes da política de ciência e tecnologia no Brasil durante a ditadura militar. Traçamos um perfil social dos dirigentes da Coordenação de Aperfeiçoamento de Pessoal de Ensino Superior (Capes), do Conselho Nacional de Desenvolvimento Científico e Tecnológico (CNPq) e da Financiadora de Estudos e Projetos (Finep) entre 1964 e 1985, considerando variáveis como sexo, região de origem, curso e universidade de formação, e atuação. Propomos uma abordagem ancorada na sociologia histórica, atenta à trajetória social dos agentes e aos conflitos do campo acadêmico-científico. A pesquisa revelou que a direção da política de ciência e tecnologia durante a ditadura foi controlada por professores homens formados em cursos profissionais tradicionais, o que aproxima seus dirigentes do perfil dos gestores das universidades brasileiras.

## Abstract (en)

This article presents a prosopography of the leaders of science and technology policy in Brazil during the Military Dictatorship. We draw the social profile of the general directors of Coordenação de Aperfeiçoamento de Pessoal de Ensino Superior (Capes), Conselho Nacional de Desenvolvimento Científico e Tecnológico (CNPq) and Financiadora de Estudos e Projetos (Finep) between 1964 and 1985, looking at variables such as gender, region of origin, course and university of training, and professional career. We proposed an approach anchored in historical sociology, analyzing the social trajectory of the agents and the conflicts in the academic-scientific field. The research revealed that the management of the science and technology policy during the dictatorship was controlled by male professors trained in traditional professional courses, a profile similar to that of the presidents of Brazilian universities.

As políticas de ciência e tecnologia, mais recentemente denominadas políticas de ciência, tecnologia e inovação, a despeito da sua importância, ainda constituem um tema relativamente marginal nos estudos sociais de ciência e tecnologia (Elzinga, Jamison, 2000). Essa marginalização relativa deve-se, em parte, ao fato de o tema ter sido predominantemente tratado por uma perspectiva instrumental, que analisa essas políticas à luz dos seus resultados, e não das suas motivações sócio-históricas. Essa perspectiva mais difundida, como toda a abordagem do tipo *problem solving*, por se preocupar excessivamente com a análise da eficiência/eficácia das políticas públicas, perde uma dimensão essencial do fenômeno, a saber, o contexto social de formulação e implementação dessas políticas, cuja compreensão contribui, inclusive, para um olhar mais crítico para os seus resultados.

Por isso, na literatura internacional, ainda são poucos os trabalhos que buscam analisar as políticas de ciência, tecnologia e inovação de um ponto de vista histórico-sociológico, o que implica reconstruir o contexto social de sua formulação e implementação, com ênfase nos atores e grupos sociais envolvidos nesse processo (Shinn, 2002; Godin, 2004, 2006; Sharif, 2006). No caso brasileiro, também não são muitos os autores que buscam analisar a formulação e implementação das políticas de ciência, tecnologia e inovação, indo além da mera análise da eficiência/eficácia dessas medidas (ver, por exemplo, Dagnino, 2007; Carlotto, 2013; Dias, 2012; Viotti, 2008).

O presente artigo se inscreve entre os trabalhos que buscam suprir essa lacuna (Dagnino, 2007; Dias, 2012; Viotti, 2008), analisando o processo de construção social das políticas de ciência, tecnologia e inovação à luz dos grupos que trabalham na sua elaboração. Nesse sentido, é parte de um esforço mais amplo de pesquisa, cujo objetivo geral é dar continuidade a tentativas anteriores de construir uma sociologia histórica das políticas de ensino superior, ciência, tecnologia e inovação no Brasil (Carlotto, 2013, 2014, 2022; Garcia, Carlotto, 2012, 2013; Carlotto, Garcia, 2018, 2021; Arbix, Toledo, 2013; Toledo, 2015, 2019, 2020).

Ao fazer isso, ancora-se também em um diálogo crítico com a historiografia hegemônica sobre a política de ciência e tecnologia, indo além da perspectiva predominantemente marcada pela noção de comunidade científica e, com ela, da primazia dos indivíduos imbuídos dos seus valores e normas. Por isso, propõe realizar uma prosopografia que apresente sociologicamente o grupo que hegemonizou a política de ciência e tecnologia durante a ditadura, destacando o papel dos conflitos inerentes às hierarquias internas do campo acadêmico-científico, ao demonstrar que esse grupo representa posições específicas de um campo, e não o todo da comunidade acadêmica. Mais especificamente, a pergunta desta pesquisa é: em que medida a formulação da política científico-tecnológica durante a ditadura militar foi hegemonizada por dirigentes que se formaram e atuaram nos campos mais básicos da ciência?

Para respondê-la, este trabalho traça o perfil social dos dirigentes das principais agências nacionais de política científico-tecnológica do país entre 1964, ano do golpe militar, e 1985, ano de criação do Ministério de Ciência e Tecnologia e início do processo de redemocratização do país. Mais concretamente, neste artigo será analisado o perfil social dos dirigentes da Coordenação de Aperfeiçoamento de Pessoal de Ensino Superior (Capes), do Conselho Nacional de Desenvolvimento Científico e Tecnológico (CNPq) e da Financiadora de Estudos e Projetos (Finep) entre 1964 e 1985. O período escolhido para este mapeamento, além de abordar um momento histórico específico, a ditadura militar, permite uma comparação entre o perfil dos dirigentes da política científico-tecnológica e o perfil dos reitores das universidades brasileiras desse mesmo período e mapeados previamente pela literatura (Carlotto, Garcia, 2021).

Do ponto de vista da sua estrutura, este trabalho divide-se em três partes, além desta introdução. Na primeira, “A política de ciência, tecnologia e inovação como problema sociológico”, desenvolvemos alguns dos pressupostos teóricos e metodológicos da nossa abordagem, procurando dialogar com a literatura que analisou as políticas de ciência, tecnologia e inovação no país. Na segunda, “Os dirigentes da política de ciência e tecnologia no Brasil entre 1964 e 1985”, apresentamos os dados levantados pela pesquisa empírica, procurando apresentar uma análise do perfil social dos dirigentes da política científica e tecnológica do país entre 1964 e 1985, comparando-os com o perfil dos dirigentes universitários do mesmo período. Nas considerações finais, além de sistematizar os principais achados, apresentamos alguns apontamentos gerais sobre desdobramentos futuros desta pesquisa.

## A política de ciência, tecnologia e inovação como problema sociológico

O campo de estudos das políticas de ciência, tecnologia (e inovação)^1^ é um daqueles domínios em que descrição, análise e prescrição se confundem, dificultando os esforços mais críticos para compreender o significado social e político da promoção estatal da ciência, da tecnologia e do desenvolvimento tecnoprodutivo em contextos históricos específicos.

A despeito dessa limitação geral, e considerando especificamente o caso brasileiro, alguns trabalhos procuraram analisar a política científico-tecnológica considerando não apenas a sua eficiência/eficácia geral, mas também o seu processo de elaboração e implementação (Burgos, 1999; Erber, Guimarães, Araújo Jr., 1985). Mais recentemente, essa tendência ganhou novo impulso com a aplicação da abordagem de “análise de política pública” para o campo das políticas de ciência, tecnologia e inovação, com destaque para os trabalhos de Renato Dagnino (2007) e Rafael de Brito Dias (2012).

Ao aplicar o enfoque de “análise de políticas” para considerar o “momento de formulação” e o “momento de implementação” das políticas de ciência, tecnologia e inovação, Renato Dagnino destaca o papel central da “comunidade científica” na definição dessa agenda. O argumento de Dagnino (2007, p.189) ganha densidade quando o autor mobiliza toda uma tradição de análise das políticas de ciência, tecnologia e inovação na América Latina que aponta, justamente, para a baixa participação do setor privado na elaboração dessa agenda:

O argumento central deste trabalho é que em nosso país (e ao que parece, em geral, na América Latina), mais do que nos países avançados, a comunidade de pesquisa possui um papel dominante na elaboração da PCT. Ou seja, que ela e em particular os professores-pesquisadores com desempenho profissional no âmbito do CPESP [Complexo Público de Ensino Superior e Pesquisa] são praticamente os únicos responsáveis não apenas pela definição da agenda de pesquisa e pela formulação da política de pesquisa, mas pelas atividades de avaliação que delas decorrem (e além disso, obviamente, pela implementação da política).

No mesmo sentido e com uma metodologia de pesquisa bastante similar, Rafael de Brito Dias (2012) procurou mostrar como, apesar das políticas de ciência, tecnologia e inovação resultarem quase sempre de uma negociação entre uma “agenda da ciência” e “uma agenda da sociedade”, “os cientistas” são sempre os principais envolvidos em sua elaboração, especialmente em países em que o Estado responde por grande parte dos investimentos em ciência, tecnologia e inovação, como é o caso do Brasil. Assim, mesmo considerando a existência de *advocacy coalitions* envolvendo diferentes setores sociais, o peso da comunidade científica acaba predominando e definindo o sentido geral das políticas de ciência, tecnologia e inovação.

Apesar de o “enfoque de análise de políticas” ter representado um avanço importante na compreensão da trajetória das políticas de ciência, tecnologia e inovação brasileiras, ao destacar o peso de um setor social específico – a assim chamada “comunidade científica” – na sua formulação e implementação, também é verdade que essa abordagem apresenta uma limitação inerente à própria centralidade que a noção de “comunidade científica” adquire para esses autores.

Isso porque, embora identifiquem segmentações internas à “comunidade científica”, essas não implicam necessariamente o reconhecimento de hierarquias e assimetrias padronizadas e socialmente ancoradas. Nesse sentido, entre os “aviadores-cientistas” que, segundo Rafael Dias (2012, p.65), hegemonizaram a política de ciência e tecnologia dos anos 1950, os “professores-engenheiros” que a controlaram na década de 1960, e os “pesquisadores extensionistas” que influenciaram os anos 1970, é difícil ir além de categorias descritivas, por meio do mapeamento de estruturas hierárquicas e espaços de conflito.

Essa crítica pode ser feita até mesmo a trabalhos que colocam centralidade na identificação de grupos e agentes empiricamente mais circunscritos, como o que procurou analisar o processo de negociação e implementação do Laboratório Nacional de Luz Síncrotron, concluindo que era possível observar uma forte influência do chamado “grupo da Unicamp” na formulação e implementação de políticas de ciência, tecnologia e particularmente inovação no país, a partir dos anos 1980 (Carlotto, 2013). Apesar de identificar a existência desse grupo, cuja nomeação era usada pelos próprios agentes entrevistados, como uma categoria “nativa”, a autora não avançou no sentido de caracterizar, sociologicamente, o perfil desse grupo, mobilizando sua nomeação numa chave puramente descritiva.

É na tentativa de ir além dessas categorias descritivas, não para abandoná-las, mas para conferir-lhes mais densidade analítica, que o presente trabalho propõe trocar o conceito de comunidade científica pelo conceito de campo acadêmico-científico (Bourdieu, 1975, 2004) e, por meio dele, identificar qual o perfil social das elites dirigentes da ciência e tecnologia no país.

Ao fazer isso, este trabalho aproxima-se de outros esforços que vêm sendo realizados nessa direção, seja para identificar quais são os saberes de Estado mais valorizados e, portanto, mais propensos a garantir aos seus detentores lugar de destaque na formulação de políticas públicas para a educação, ciência, tecnologia e desenvolvimento (Almeida, 2008; Carlotto, 2013; Carlotto, Garcia, 2018, 2021; Garcia, Carlotto, 2013; Sharif, 2006; Godin, 2006), seja para definir o perfil social das elites científicas e dirigentes do país (Hey, 2008; Hey, Catani, 2006).

Vale notar que, ao priorizar uma abordagem histórico-sociológica, que enfatiza a importância do perfil social dos dirigentes das principais agências de política de ciência e tecnologia do país, busca-se operar um duplo deslocamento. Por um lado, queremos mostrar que não basta destacar, como já foi feito na literatura (Dagnino, 2007; Dias, 2012; Carlotto, 2013; Schwartzman, 1979; Viotti, 2008), o papel central da “comunidade científica” – quase sempre analisada como grupo social relativamente homogêneo e coeso. Também é preciso entender as disputas internas ao campo científico – ou, para sermos mais precisos, do campo acadêmico-científico^2^ –, altamente heterogêneo e hierarquizado e, como tal, permeado por assimetrias e conflitos (Bourdieu, 1975, 2002). Por outro lado, essa perspectiva opera um segundo deslocamento, ao reorientar o foco da ação de indivíduos, quase sempre tratados numa chave heroica, para o papel dos grupos protagonistas dos embates em torno da orientação das políticas nacionais de ciência, tecnologia e inovação e suas concepções sobre critérios de certificação científica, legitimidade acadêmica e desenvolvimento tecnológico (Dagnino, 2007; Dias, 2012; Carlotto, 2013; Schwartzman, 1979).

Esse duplo deslocamento distancia o presente trabalho da perspectiva predominante na historiografia sobre instituições acadêmicas, científicas e, por conseguinte, sobre política científico-tecnológica, marcada por um esforço de reconstrução histórica pautado por uma visão excessivamente institucional (Carlotto, 2022). Essa historiografia, que acaba aproximando-se do que podemos classificar como uma “história oficial”, opera, de um lado, para minimizar, quando não invisibilizar, os conflitos internos a essas instituições e políticas e, de outro, para superdimensionar o papel de indivíduos, concedendo demais a *illusio* do campo acadêmico, onde, segundo Bourdieu (1984), tudo passa por construir “um nome”. É isso que explica por que trabalhos amplamente difundidos sobre a história da política de ciência e tecnologia nacional, escritos em geral por encomenda das próprias agências e instituições de fomento, ancoram-se na noção de comunidade e se baseiam na metodologia da história oral que prioriza o testemunho pessoal (Ferreira, Moreira, 2002; Lacaz, Mazzieri, 1995; Lima, Fonseca, Santos, 2004; Martins, Barbuy, 1998; Motoyama, 1999, 2002, 2006; Nakata, 2007; Schwartzaman, 1979).

Na contramão dessas tendências, fazemos um esforço para objetivar trajetórias e perfis individuais na tentativa de despersonalizar a história dessas agências e instituições, transformando indivíduos empíricos em atores sociologicamente construídos. Isso implica romper com a história oral como modelo de reconstrução histórica institucional para assumir uma perspectiva de sociologia histórica estrutural em que as propriedades sociais, institucionais e profissionais vêm para o primeiro plano (Carlotto, 2022). Nesse sentido, metodologicamente, vale frisar que a prosopografia – a biografia coletiva de um grupo baseada em atributos sociais (Charle, 2006) – é um método que permite ir além da reconstrução histórica baseada na idealização da unidade da comunidade científica e no testemunho pessoal de dirigentes que falam a partir de posições de poder institucional. Ao romper com essa perspectiva, esperamos jogar luz sobre os processos sociais que concorrem para construir um falso universal, em que o todo – a comunidade científica – é forjado pela parte – setores politicamente hegemônicos do campo acadêmico-científico, que pautam critérios de validade, rigor e cientificidade.

É importante frisar, ainda, que a investigação sistemática das origens sociais e da trajetória dos dirigentes das instituições de ciência, tecnologia e inovação no Brasil da segunda metade do século passado até o presente é relativamente escassa na literatura especializada. Em linha com o que destacamos até aqui, estudos sobre instituições específicas (Lima, Pinto, 2003; Hamilton, Fonseca, 2003) ou trajetórias individuais (Lima, 2003) têm predominado na produção científica, não obstante a existência de trabalhos que adotam uma visão de conjunto das políticas e instituições de ciência, tecnologia e inovação em período mais amplo (como Dagnino, 2007; Viotti, 2008; Dias, 2012). Este artigo avança na caracterização das origens sociais e trajetórias dos dirigentes das principais instituições de fomento à ciência e à tecnologia no Brasil, pensados como grupo, preenchendo com isso importante hiato da bibliografia sobre o assunto, que ora singulariza instituições e dirigentes (mediado por noções tais como “a instituição”, “o dirigente”, “a cientista”), ora homogeneíza essas mesmas instituições e dirigentes (por meio de conceitos como “a política de CTI”, “as instituições de CTI”, “a elite científica”, “a comunidade acadêmica”).

Por fim, o presente artigo, ancorado na sociologia histórica e visando superar os limites identificados na literatura dominante sobre a política de ciência e tecnologia brasileira, coloca-se no terreno do debate interdisciplinar entre a sociologia e a história. Isso implica aceitar interpelações críticas de lado a lado e, ao mesmo tempo, apostar no potencial que essas interpelações têm para ambas as disciplinas, mesmo que esse esforço seja permeado de estranhamentos teóricos e metodológicos de parte a parte.

## Os dirigentes da política de ciência e tecnologia no Brasil entre 1964 e 1985

Para analisar o perfil social de quem dirigiu a política científico-tecnológica brasileira entre 1964 e 1985, mapeamos os ocupantes dos cargos máximos das três principais agências de fomento da época: a Coordenação de Aperfeiçoamento de Pessoal de Ensino Superior (Capes), fundada em 1951; o Conselho Nacional de Pesquisas (CNPq), atualmente denominado Conselho Nacional de Desenvolvimento Científico e Tecnológico, fundado também em 1951; e a Financiadora de Estudos e Projetos (Finep), criada em 1967. Vale notar que até 1985 inexistia uma estrutura burocrática que centralizasse a política de ciência e tecnologia, o que aumenta a importância dessas agências. O próprio Ministério de Ciência e Tecnologia só foi criado em 1985, no contexto da redemocratização.

Nessa etapa, concentramos nosso levantamento nos dirigentes máximos das instituições apontadas (isto é, seus presidentes e diretores executivos), nomeados entre 1964 e 1985, considerando as seguintes variáveis: curso de formação; universidade de formação; sexo; estado de origem; instituição em que atuava no momento da nomeação. O foco em propriedades ligadas à trajetória profissional e acadêmica tem raízes teóricas e metodológicas. Teoricamente, a pergunta essencial da pesquisa se refere às assimetrias e conflitos inerentes ao campo acadêmico-científico, daí o interesse principal recair sobre a posição desses agentes nesse campo, que não é apenas científico no sentido estrito, mas engloba setores acadêmicos mais amplos, como veremos. Metodologicamente, esses são os dados disponíveis nas principais fontes consultadas, sendo as propriedades sociais, tais como origem social e escolaridade dos pais, não só de mais difícil acesso como também relativamente indiferentes para a hipótese de que partimos.

Nesse sentido, do ponto de vista da coleta dos dados, construímos um banco de dados que foi completado por pesquisa documental em bases de dados consultadas nesta ordem: (1) plataforma Currículos Lattes; (2) Dicionário Histórico-biográfico Brasileiro, do CPDOC/FGV; (3) Arquivo Histórico da Capes; (4) Centro de memória CNPq; (5) seção histórica do portal Finep; (6) buscas gerais na internet visando complementar ou checar informações. O resultado desse mapeamento é apresentado em seguida, primeiro por agência e, a seguir, considerando o conjunto dos dirigentes que comandaram as três agências analisadas. Ressalte-se que o objetivo deste artigo é mapear sociologicamente os grupos que compuseram a direção dessas instituições, chamando a atenção para sua especificidade em comparação com o conjunto do campo acadêmico-científico. Nesse sentido, avaliar as políticas implementadas por esses agentes no período em questão, embora relevante, escapa aos objetivos do presente trabalho.

## Coordenação de Aperfeiçoamento de Pessoal de Ensino Superior

A Capes foi criada em 1951, em um contexto em que as políticas de ciência e tecnologia se institucionalizavam em todo mundo por meio de agências de financiamento de projetos específicos e de formação de recursos humanos (Elzinga, Jamison, 2000). Originalmente, a Capes organizou-se como uma campanha ligada ao então Ministério de Educação e Saúde (posteriormente, Ministério de Educação e Cultura, MEC) para a concessão de bolsas visando ao aperfeiçoamento de pessoal de ensino superior. Em 1961, a campanha foi incorporada diretamente à Presidência da República, ali permanecendo até 1964, quando a Capes retornou ao MEC.

A partir desse retorno para o MEC, a primeira fase da ditadura militar, em especial o período que vai de 1964 a 1969, foi bastante conturbado para a Capes. A demissão de Anísio Teixeira em 1964, seu idealizador e primeiro presidente por quase 15 anos (1951-1964), e sua substituição por Susana Gonçalves, contraparente do general Castelo Branco e funcionária da reitoria da Pontifícia Universidade Católica do Rio de Janeiro (PUC-Rio), deu início a uma fase marcada por elevado número de substituição de dirigentes. Assim, em cinco anos (1964-1969), a Capes teve cinco presidentes: Susana Gonçalves, Gastão Dias Velloso, Mário Werneck de Almeida Lima, Nelson Afonso do Valle Silva e Jéferson Andrade Machado de Góis Soares, esses dois últimos provavelmente funcionários da instituição sobre os quais temos poucas informações.

A partir de 1970, com a nomeação de Celso Barroso Leite, os dirigentes da instituição passaram a ficar mais tempo no cargo. Entre 1970 e 1989, foram quatro presidentes: Celso Barroso Leite (1970-1974), Darcy Closs (1974-1979), Cláudio de Moura Castro (1979-1982) e, o mais longevo de todos, Edson Machado de Sousa (1982-1989). Assim, se entre 1964 e 1969 cada dirigente ficou em média pouco mais de um ano à frente da instituição, no período de 1970 a 1989, os dirigentes ficaram em média quase cinco anos à frente da agência. As implicações dos diferentes padrões de estabilidade de dirigentes sobre o desenvolvimento institucional e a atuação da Capes, que não discutiremos neste trabalho, são fáceis de imaginar. De todo modo, a estabilização da gestão da Capes corresponde ao período de consolidação da reforma universitária de 1968, quando, seguindo o modelo estadunidense, o país passou a implementar um efetivo sistema nacional de pós-graduação, cuja avaliação ficou a cargo da instituição, papel que cumpre até hoje (Castro, Soares, 1983).

É importante frisar que o modelo de pós-graduação implementado no Brasil foi desenhado sobretudo a partir do chamado “Relatório Sucupira”, parecer da Comissão Federal de Educação de 3 de dezembro de 1965, assinado por Newton Sucupira, relator, e demais membros do grupo de trabalho, a saber, Clóvis Salgado, José Barreto Filho, Alceu Amoroso Lima, Anísio Teixeira, Valnir Chagas e Rubens Maciel. Apesar do “Relatório Sucupira” ter sido publicado em 1965, sua implementação efetiva é posterior à Reforma de 1968, de modo que a Capes só foi efetivamente associada à criação do sistema nacional de pós-graduação no período subsequente à reforma.

O foco deste artigo, porém, não são as políticas desenhadas e implementadas durante a ditadura, incluindo o sistema de avaliação, mas, sim, o perfil social desses dirigentes, construído a partir de algumas variáveis-chave, ligadas à sua trajetória acadêmica e profissional. O Quadro 1 apresenta uma síntese do perfil social dos dirigentes da Capes nomeados entre 1964 e 1985.

**Quadro 1:** : Dirigentes da Capes nomeados entre 1964 e 1985

Dirigente	Período à frente da Capes	Curso de formação	Universidade de formação	Instituição em que atuava antes de assumir o órgão
Susana Gonçalves	1964-1966	Não identificado	Não identificado	Reitoria da Pontifícia Universidade Católica do Rio de Janeiro (PUC-Rio)
Gastão Dias Velloso	1966-1967	Medicina	Universidade Federal de Minas Gerais (UFMG)	Universidade Federal do Rio de Janeiro (UFRJ)
Mário Werneck de Almeida Lima	1967-1968	Engenharia	Universidade Federal de Minas Gerais (UFMG)	Universidade Federal de Minas Gerais (UFMG)
Nelson Afonso do Valle Silva	1969	Não identificado	Não identificado	Não identificado
Jéferson Andrade Machado de Góis Soares	1969	Não identificado	Não identificado	Não identificado
Celso Barroso Leite	1970-1974	Direito; Jornalismo	Universidade Federal Fluminense (UFF); Universidade Federal do Rio de Janeiro (UFRJ)	Capes
Darcy Closs	1974-1979	Geociências	Universdade Federal do Rio Grande do Sul (UFRGS)	Universdade Federal do Rio Grande do Sul (UFRGS)
Cláudio de Moura Castro	1979-1982	Economia	Universidade Federal de Minas Gerais (UFMG)	Instituto de Estudos Avançados de Educação/Fundação Getulio Vargas
Edson Machado de Sousa	1982-1989	Matemática	Universidade Federal do Paraná (UFPR)	Secretaria Estadual de Educação do Paraná

Fonte: pesquisa documental e elaboração próprias.

O primeiro aspecto que salta aos olhos é a presença de apenas uma mulher à frente da Capes no período em análise: Susana Gonçalves (1964-1966). Aliás, dos 21 dirigentes das três instituições que estamos analisando (Capes, CNPq e Finep), Susana Gonçalves figura como única mulher, representando, em termos proporcionais, menos de 5% de nossa amostra.

Dando sequência à análise, a pesquisa documental não encontrou dados completos sobre três dos nove dirigentes da Capes no período: Susana Gonçalves, Nelson Afonso do Valle Silva e Jéferson Andrade Machado de Góis Soares. Dos seis dirigentes para os quais foram encontrados dados sobre curso de formação, universidade de formação e instituição em que atuavam antes de assumir o órgão – Gastão Dias Velloso, Mário Werneck de Almeida Lima, Celso Barroso Leite, Darcy Closs, Cláudio de Moura Castro e Edson Machado de Sousa –, um era formado em medicina, um em engenharia, um em direito e jornalismo, um em geociências e um em economia.

Embora a variação dos cursos de formação pareça grande, sugerindo a ausência de um padrão em relação à área de formação desses dirigentes, considerando a análise desenvolvida em outros trabalhos (Carlotto, 2014; Carlotto, Garcia, 2021), podemos concluir que uma parte importante deles era formada em cursos “profissionais tradicionais”, como direito, medicina, engenharia e economia.^3^

Nesse sentido, a análise dos dirigentes máximos da Capes já indica que a nossa hipótese central, de que a formulação da política científico-tecnológica seria hegemonizada por profissionais oriundos dos campos mais básicos da ciência, precisa ser repensada. Mesmo considerando os dois dirigentes formados em matemática e estatística, ambos trabalhavam com a produção de indicadores e, portanto, associados ao desenho dos primeiros sistemas de mensuração e avaliação dos investimentos em educação superior, ciência e tecnologia (Almeida, 2008; Carlotto, 2013), estando, por isso mesmo, mais ligados à área de economia e administração pública do que de matemática propriamente dita.

Entre os seis dirigentes para os quais dispomos de dados completos, três se formaram na Universidade Federal de Minas Gerais (UFMG), um na Universidade Federal do Rio Grande do Sul (UFRGS) e um na Universidade Federal do Paraná (UFPR). Isso indica que a Capes teve, nesse período, dirigentes oriundos sobretudo das regiões Sul e Sudeste, e particularmente do sistema federal de ensino, o que se mostrará relevante quando comparado ao padrão do CNPq, hegemonizado pelo Nordeste, e da Finep, muito mais atrelada ao Sudeste. Em termos mais específicos, a relação entre a Capes e a UFMG merece um aprofundamento futuro, mas uma hipótese que já pode ser levantada é que a montagem do sistema de avaliação da Capes, nos anos 1970, envolveu o domínio de conhecimentos e técnicas que eram predominantes na instituição.

Considerando a instituição em que esses dirigentes atuavam no momento da nomeação – um primeiro, mas significativo, elemento dessas trajetórias –, merece destaque o fato de que quatro tinham atuação em universidades antes de assumirem a direção da Capes (Universidade Federal do Rio de Janeiro, UFMG, UFRGS e Fundação Getulio Vargas-Rio), aos quais podemos acrescentar Susana Gonçalves, assessora da reitoria da PUC-Rio. Por outro lado, apenas dois estavam atuando em órgãos estatais (a própria Capes e a Secretaria Estadual de Educação do Paraná). Esse dado parece indicar que a Capes teve, nesse período, um padrão de recrutamento de dirigentes atrelado às hierarquias do sistema de ensino superior do país que, como evidenciado na análise do perfil dos reitores das universidades brasileiras entre 1966, ano de fundação do Conselho de Reitores das Universidades Brasileiras, e 1985, ano de inflexão da política desse conselho, era hegemonizado por reitores igualmente oriundos do polo profissional tradicional do ensino superior, com destaque para os formados em direito (25%), medicina (23%) e engenharia (16%) (Carlotto, Garcia, 2021, p.79). Vale notar que esse padrão se assemelha muito ao do perfil dos altos dirigentes da Universidade de São Paulo (USP):

Nos dois casos, observamos não só um forte predomínio do polo profissional tradicional – em ambos os casos, maior do que 70% – como a ordem dos cursos com maior número de reitores formados é a mesma, a saber: Direito, Medicina e Engenharia e, com menor destaque, Economia, seguidas de outras profissões tradicionais de menor presença relativa, como Odontologia, Farmácia, Arquitetura e Medicina Veterinária, aqui reunidas na categoria ‘outras profissões’ (Carlotto, Garcia, 2021, p.79).

Esse perfil, com algumas inflexões, também vai marcar os dirigentes do CNPq, analisados a seguir.

## Conselho Nacional de Desenvolvimento Científico e Tecnológico

O CNPq foi criado em 1951, por iniciativa dos militares em associação com cientistas ligados, sobretudo, à física, especialmente à física atômica (Forjaz, 1989; Domingos, 2004; Motoyama, 2002). Depois de um período de grandes investimentos em áreas estratégicas entre 1951 e 1954, sob o governo Juscelino Kubistchek, o CNPq viveu uma crise ligada ao seu esvaziamento e subinvestimento. Foi somente na ditadura militar que o CNPq voltou a consolidar-se como órgão central de fomento à ciência e à tecnologia (Domingos, 2004).

Entre 1964 e 1986, o CNPq teve seis dirigentes nomeados, todos homens (Quadro 2). Com um período médio de duração de pouco mais de três anos e meio, os mandatos da cúpula do CNPq na ditadura têm duração média superior à da primeira fase da Capes (1964-1969, pouco mais de um ano à frente da instituição de cada um de seus dirigentes), mas inferior à da segunda fase daquela instituição (1970-1989, com uma média de permanência à frente da instituição de quase cinco anos).

**Quadro 2:** : Dirigentes do CNPq nomeados entre 1964 e 1985

Dirigente	Período à frente do CNPq	Curso de formação	Universidade de formação	Instituição em que atuava antes de assumir o órgão
Antônio Moreira Couceiro	1964-1970	Medicina	Faculdade de Medicina do Recife (UFPE)	Academia Brasileira de Ciências (ABC)
Arthur Mascarenhas Façanha	1970-1974	Engenharia	Instituto Militar de Engenharia (IME)	Instituto Militar de Engenharia (IME)
José Dion de Melo Teles	1975-1979	Engenharia	Instituto Tecnológico da Aeronáutica (ITA)	Departamento de Administração/UnB
Maurício Matos Peixoto	1979-1980	Engenharia	Escola Nacional de Engenharia (UFRJ)	Instituto de Matemática e Estatística/USP
Lynaldo Cavalcanti de Albuquerque	1980-1985	Engenharia	Universidade Federal de Pernambuco (UFPE)	Universidade Federal da Paraíba (UFPB)
Roberto Figueira Santos	1985-1986	Medicina	Universidade Federal da Bahia (UFBA)	Universidade Federal da Bahia (UFBA)

Fonte: pesquisa documental e elaboração próprias.

Ao contrário do que se observa no caso da Capes, os dirigentes do CNPq foram todos, sem exceção, recrutados do meio universitário, inclusive o general de brigada Arthur Mascarenhas Façanha, professor do Instituto Militar de Engenharia (IME) e único militar à frente da instituição no período. Os dirigentes do CNPq durante a ditadura eram formados essencialmente em dois cursos superiores: engenharia (Arthur Mascarenhas Façanha, José Dion de Melo Teles, Maurício Matos Peixoto e Lynaldo Cavalcanti Albuquerque) e medicina (Antônio Moreira Couceiro e Roberto Figueira Santos).

Mais uma vez, destaca-se a hegemonia de dirigentes oriundos do campo “profissional tradicional” de ensino superior, presente também no CNPq. Note-se que dizer que esses dirigentes são oriundos de profissões tradicionais não implica negar a sua provável dedicação à atividade científico-tecnológica, mas reconhecer a existência de uma trajetória-padrão que diz muito sobre a heterogeneidade e as assimetrias da “comunidade científica”. Reforça-se a percepção de que estamos diante de um campo cujas hierarquias são evidentes, especialmente em favor de cursos profissionais tradicionais, o que se manifesta tanto no interior das instituições de ensino superior (Carlotto, 2018) quanto da política de ciência, tecnologia e inovação.

Considerando suas universidades de origem, enquanto na Capes havia a presença de pelo menos uma dirigente oriunda de universidade privada, no caso do CNPq, destaca-se o fato de que o perfil é exclusivamente de profissionais oriundos do sistema público de ensino superior de quatro diferentes estados: Rio de Janeiro (dois engenheiros, um formado pela hoje denominada Universidade Federal do Rio de Janeiro e outro pelo Instituto Militar de Engenharia), Pernambuco (um médico e um engenheiro, ambos formados pela hoje Universidade Federal de Pernambuco), São Paulo (engenheiro pelo Instituto Tecnológico da Aeronáutica) e Bahia (médico pela Universidade Federal da Bahia). Os seis dirigentes tinham atuação anterior no meio universitário.

No caso do CNPq – órgão criado, assim como a Capes, em 1951, quando a institucionalização da PCT no país passava por uma aliança entre elites científicas e militares em torno do projeto de incorporação da tecnologia nuclear (Toledo, 2020) –, fica evidente a recorrência de um padrão ainda mais homogêneo de recrutamento dos seus quadros dirigentes: homens, brancos, engenheiros ou médicos, todos com trajetória profissional em instituições públicas de ensino superior anterior à sua passagem pelo CNPq. Outro padrão, que veremos na parte final deste artigo, é a região de origem: todos, sem exceção, são nascidos no Nordeste, mesmo os formados em instituições do Sudeste do país. Esse perfil específico ganha maior significado quando comparado ao perfil dos dirigentes da Finep.

## Financiadora de Estudos e Projetos

A Finep foi criada em 1965, por intermédio de Roberto de Oliveira Campos, ministro do Planejamento. Campos diagnosticara uma carência no país de fontes de financiamento para as fases iniciais de empreendimentos econômicos (Ferrari, 2002). No entanto, o modelo de fundo não durou muito, e, dois anos depois, em 1967, a Finep tornou-se uma empresa pública, responsabilizando-se pela secretaria-executiva do Fundo Nacional de Desenvolvimento Científico e Tecnológico (FNDCT). O surgimento da Finep e do FNDCT remete a um contexto em que, não obstante as ações sistemáticas conduzidas pela Capes, pelo CNPq e pelo então Fundo de Desenvolvimento Técnico-Científico, predominava um diagnóstico de que o sistema nacional de ciência e tecnologia ainda carecia de instrumentos capazes de articulá-lo com as necessidades efetivas da economia brasileira (Ferrari, 2002).

Assim, a origem da Finep está mais ligada à tradição desenvolvimentista da burocracia estatal brasileira – não por acaso a Finep e o FNDCT foram, por muito tempo, ligados contabilmente ao Banco Nacional de Desenvolvimento Econômico, posteriormente Banco Nacional de Desenvolvimento Econômico e Social (BNDES) – do que aos esforços de financiamento sistemático da atividade científico-tecnológica por si mesma. Nesse sentido, a Finep faz parte de um momento da política de ciência, tecnologia e inovação brasileira em que essa se caracterizava pela busca sistemática de autonomia científico-tecnológica como parte nodal de um projeto nacional de desenvolvimento (Adler, 1987; Carlotto, Hitner, 2018; Toledo 2019, 2020). Essa origem específica se reflete claramente no perfil dos dirigentes máximos da instituição, nomeados a partir 1967, quando a Finep deixa de ser um fundo para se tornar um órgão estatal, sob a forma jurídico-institucional de empresa pública.

Em primeiro lugar, todos os dirigentes da Finep cuja trajetória pôde ser reconstruída na pesquisa aqui descrita atuavam, no momento da sua nomeação, não no sistema de ensino e pesquisa, como no caso da Capes e do CNPq, mas no aparelho de Estado, em especial no Ministério do Planejamento, no BNDES ou na própria Finep, órgãos que parecem formar um complexo de agências de planejamento e instrumentos de incentivo ao desenvolvimento. Mais do que acadêmicos, os dirigentes da Finep parecem ter um perfil de “técnicos do Estado”.

Outra característica dos dirigentes da Finep é que todos, sem exceção, foram formados em instituições da região Sudeste (Quadro 3). O perfil dos cursos de origem, porém, não varia muito, sendo, de novo, profissionais tradicionais: engenharia, economia e estatística. Esse padrão de formação sugere um perfil muito específico, de profissionais “tradicionais”, mas com competências de planejamento econômico e gestão.

**Quadro 3:** : Dirigentes da Finep nomeados entre 1968 e 1985

Dirigente	Período à frente da Finep	Curso de formação	Universidade de formação	Instituição em que atuava antes de assumir o órgão
Francisco Manoel de Mello Franco	1967-1971	Engenharia	PUC-Rio	Ministério do Planejamento
José Pelúcio Ferreira	1971-1979	Economia	Universidade do Distrito Federal (atual Universidade do Estado do Rio de Janeiro)	BNDES
Alfredo Luiz Baumgarten Júnior	1979-1979	Economia	Não identificado	Não identificado
Gerson Edson Ferreira Filho	1980-1983	Estatística	Escola Nacional de Ciências Estatísticas	Não identificado
José Walter Merlo	1983-1984	Engenharia	Escola Politécnica/USP	Ministério de Minas e Energia
Fabio Celso de Macedo Soares Guimarães	1985-1988	Engenharia	Não identificado	Finep

Fonte: pesquisa documental e elaboração próprias.

Assim, a despeito dessas especificidades da Finep – em especial o fato de ela ser mais ligada ao aparelho de Estado do que à estrutura acadêmico-científica do país –, existem outros traços que coincidem com o perfil geral: todos os dirigentes da Finep no período analisado são homens; praticamente todos são oriundos do chamado polo profissional tradicional, com destaque para os formados nas engenharias.

## Considerações finais

Depois de analisar separadamente o perfil dos dirigentes máximos das três principais agências federais de financiamento e formulação da política científico-tecnológica – a Capes, o CNPq e a Finep – no período 1964-1985, considerando variáveis como o curso de formação, a universidade de origem e a instituição de atuação, concluímos o artigo analisando conjuntamente esses dirigentes na tentativa de traçar um primeiro perfil das elites dirigentes da política de ciência, tecnologia e inovação brasileira durante a ditadura militar.

O Quadro 4 sintetiza algumas informações levantadas a respeito dos 21 dirigentes que compõem a nossa amostra. A partir dela podemos traçar um primeiro perfil prosopográfico da elite dirigente da PCT brasileira entre 1964 e 1985.

**Quadro 4:** : Dirigentes máximos da política científico-tecnológica brasileira nomeados entre 1964 e 1985

Dirigente	Órgão	Sexo	Curso de origem	Perfil do curso de origem	Estado de origem	Instituição do momento da nomeação	Perfil da nomeação
Susana Gonçalves	Capes	F	Não identificado	Não identificado	Minas Gerais	PUC-Rio	Administração universitária
Gastão Dias Velloso	Capes	M	Medicina	Profissional tradicional	Minas Gerais	UFRJ	Academia
Mário Werneck de Almeida Lima	Capes	M	Engenharia	Profissional tradicional	Minas Gerais	UFMG	Academia
Nelson Afonso do Valle Silva	Capes	M	Não identificado	Não identificado	Não identificado	Não identificado	Não identificado
Jéferson Andrade Machado de Góis Soares	Capes	M	Não identificado	Não identificado	Não identificado	Não identificado	Não identificado
Celso Barroso Leite	Capes	M	Direito; Jornalismo	Profissional tradicional	Não identificado	Capes	Burocracia estatal
Darcy Closs	Capes	M	Geociências	Acadêmico-científico	Rio Grande do Sul	UFRGS	Academia
Cláudio de Moura Castro	Capes	M	Economia	Profissional tradicional	Rio de Janeiro	IEAE/FGV	Administração universitária; Academia
Edson Machado de Sousa	Capes	M	Matemática	Acadêmico-científico	Paraná	Secretaria Estadual de Educação do Paraná	Burocracia estatal; Administração universitária
Antônio Moreira Couceiro	CNPq	M	Medicina	Profissional tradicional	Pernambuco	ABC	Academia
Arthur Mascarenhas Façanha	CNPq	M	Engenharia	Profissional tradicional	Ceará	IME	Militar; Academia
José Dion de Melo Teles	CNPq	M	Engenharia	Profissional tradicional	Piauí	UnB	Militar; Academia
Maurício Matos Peixoto	CNPq	M	Engenharia	Profissional tradicional	Ceará	Instituto de Matemática e Estatística/USP	Academia
Lynaldo Cavalcanti de Albuquerque	CNPq	M	Engenharia	Profissional tradicional	Pernambuco	UFPB	Administração universitária; Academia
Roberto Figueira Santos	CNPq	M	Medicina	Profissional tradicional	Bahia	UFBA	Administração universitária; Academia
Francisco Manoel de Mello Franco	Finep	M	Engenharia	Profissional tradicional	Rio de Janeiro	Ministério do Planejamento	Burocracia estatal
José Pelúcio Ferreira	Finep	M	Economia	Profissional tradicional	Minas Gerais	BNDES	Burocracia estatal
Alfredo Luiz Baumgarten Júnior	Finep	M	Economia	Profissional tradicional	Não identificado	Não identificado	Não identificado
Gerson Edson Ferreira Filho	Finep	M	Estatística	Acadêmico-científico	Não identificado	Não identificado	Não identificado
José Walter Merlo	Finep	M	Engenharia	Profissional tradicional	São Paulo	Ministério de Minas e Energia	Burocracia estatal
Fabio Celso de Macedo Soares Guimarães	Finep	M	Engenharia	Profissional tradicional	Rio de Janeiro	Finep	Burocracia estatal

Fonte: pesquisa documental e elaboração próprias.

A primeira dimensão relevante, anteriormente comentada e já esperada, é o perfil predominantemente masculino dos dirigentes máximos da PCT no país durante a ditadura. Embora esse resultado fosse esperado, é importante ressaltá-lo para escapar a certa naturalização da exclusão das mulheres da gestão científico-tecnológica, inerente à ideia de que a ausência de mulheres nesse âmbito deve-se, na verdade, à pouca participação delas na ciência e tecnologia como um todo. De fato, num primeiro momento, é possível supor que o baixo percentual de mulheres na amostra reflete, na verdade, a baixa presença, naquela época, de mulheres na educação superior como um todo, resultando de maneira “natural” na quase completa ausência de mulheres entre os dirigentes da política científico-tecnológica na ditadura. Essa suposição precisa, no entanto, ser mais bem qualificada. Como aponta a análise de Moema de Castro Guedes (2008) sobre a presença feminina no ensino superior, as mulheres já vinham ingressando no sistema universitário do país desde a década de 1940, processo que se intensificou nos anos 1960 e 1970, em função da equiparação entre o curso normal e científico para fins de ingresso no ensino superior.

Como consequência, no Censo Demográfico de 1980, a população feminina com ensino superior já superava a população masculina com o mesmo nível de escolaridade, o que permite sustentar que as mulheres já representavam, no final da década de 1970, boa parte dos egressos de curso superior do país (Guedes, 2008, p.122). Se, por um lado, é verdade que a presença das mulheres no ensino superior, até hoje, tende a se concentrar em certas áreas, num processo de guetização ou, se quisermos, de segregação horizontal (Ávila, Portes, 2009), também é verdade que essa segmentação contribui tanto para invisibilizar a presença das mulheres na ciência e na universidade como um todo (Costa, 2006; Ferreira et al., 2008) como para estruturar hierarquias internas às áreas ligadas à segregação de gênero (Bourdieu, 2002). Em outras palavras, é o caráter majoritariamente masculino das áreas “duras” da ciência, ou, no caso brasileiro, dos cursos profissionais tradicionais em hierarquia análoga, que ajuda a explicar por que são essas áreas que hegemonizam a estrutura de comando tanto da política de ciência e tecnologia quanto do poder universitário (Carlotto, 2014, 2018).

É relevante apontar que, em um levantamento feito em 2023 (Moreira, Carlotto, 2023), considerando todos os dirigentes da Capes, CNPq e Finep entre 1951 e 2022, foi observado que apenas a Capes contou com mulheres entre seus dirigentes, enquanto a Finep e o CNPq nunca foram dirigidos por mulheres, mesmo depois da redemocratização e mesmo sendo elas, desde os anos 1980, maioria no ensino superior do país. Significa dizer, em suma, que falar de dirigentes da ciência na ditadura é falar essencialmente de homens dirigentes, ou, para sermos ainda mais precisos: homens brancos dirigentes, sendo que, depois da ditadura, o perfil não mudou essencialmente.

Por isso, de novo, nos interessa frisar que existe uma hierarquização do campo acadêmico-científico que está fortemente ligada às desigualdades de gênero: a presença das mulheres se reduz à medida que se avança nos níveis hierárquicos da carreira, sendo baixíssima – quando não, nula – nos postos máximos de poder. Mas, para além disso, as hierarquias que separam as diferentes áreas do conhecimento e que pautam, como mostramos aqui, o controle da política de ciência e tecnologia (e inovação) envolvem também uma dimensão de gênero, como mostram dados recentes:

Em 2016, as mulheres eram maioria em todos os níveis de treinamento pelo CNPq. Na graduação, eram 60%. No mestrado *stricto sensu*, eram 58,9%. No doutorado, 56,3%. Esses dados poderiam ser interpretados como uma superação dos desafios de gênero na carreira acadêmica, entretanto, ao analisar a condição de liderança entre os sexos masculino e feminino, é possível notar que há uma vantagem masculina histórica e significativa ... Além disso, os desafios das mulheres também partem da ‘territorialidade’ do conhecimento. Ao analisar as grandes áreas do conhecimento, pode-se observar que os homens predominam nas Engenharias; nas Ciências Exatas e da Terra, nas Ciências Agrárias e nas Ciências Sociais Aplicadas. Em contrapartida, as mulheres são maioria nas áreas de Linguística, Letras e Artes, Ciências Humanas, Ciências da Saúde e Ciências Biológicas (Boechat, Carlotto, 2017, p.4-5).

Essa dupla segregação (vertical e horizontal) é um traço constitutivo do campo acadêmico-científico brasileiro, embora seja também observado em outros países. Assim, se compararmos esses dados com os dos dirigentes da USP (Carlotto, 2014) e de outras universidades brasileiras (Carlotto, Garcia, 2021), nota-se que o mesmo padrão, marcadamente masculino, está associado ao predomínio de áreas específicas do conhecimento na direção da gestão universitária – e, como mostramos aqui, da política científico-tecnológica – durante a ditadura militar.

Nesse sentido, outra dimensão que merece destaque é o perfil do curso de graduação realizado pelos dirigentes máximos da PCT. Como já enfatizado, além de um forte predomínio de cursos ligados ao polo profissional tradicional do campo acadêmico, destacam-se entre esses os cursos de engenharia, medicina e economia. Segundo nosso levantamento, dos 21 dirigentes, oito (38%) são formados em engenharia, dois (14%) em economia, dois (14%) em medicina e apenas um em direito. Se traduzirmos essas informações em termos da polaridade essencial do campo acadêmico-científico brasileiro, temos que, dos 21 dirigentes da PCT durante a ditadura, 15 (aproximadamente 72%) são oriundos do polo profissional tradicional do campo acadêmico, ao passo que apenas dois (14%) vêm explicitamente do polo acadêmico-científico.

Isso nos permite afirmar que o perfil dos dirigentes da política de ciência e tecnologia brasileira ao mesmo tempo se aproxima e se afasta do perfil dos reitores das universidades brasileiras durante o mesmo período. Se aproxima na medida em que ambos são marcados pelo predomínio de egressos de cursos profissionais mais tradicionais, e se afasta porque, enquanto as universidades brasileiras tinham, entre seus reitores, quase sempre advogados e médicos, a política de ciência e tecnologia parece ser hegemonizada por engenheiros e, em menor medida, economistas e médicos. Isso sugere que, se existia uma “divisão social do trabalho de direção” entre o ensino superior e a PCT, ela não se dava entre profissionais e cientistas, como imaginávamos, mas dentro do próprio polo profissional tradicional entre um perfil mais bacharelesco controlando as universidades e um perfil mais técnico controlando a PCT.

Do ponto de vista analítico proposto por este artigo, porém, é importante explicitar que o predomínio do polo profissional tradicional entre os dirigentes das políticas universitárias e de ciência e tecnologia durante a ditadura militar é indissociável do fato de que, comparativamente ao resto do campo, constituído basicamente pelas Faculdades de Educação e Filosofia, Ciências e Letras desmembradas após a reforma universitária de 1968, esse era o polo mais elitizado e mais masculino do campo acadêmico-científico como um todo (Miceli, 1989; Limongi, 1989; Carlotto, 2014). Nesse sentido, não é irrelevante que esse polo tenha controlado, igualmente, a PCT durante a ditadura, cabendo perguntar se isso muda a partir da redemocratização.

É importante frisar que, mesmo considerando apenas o curso de formação de graduação dos dirigentes, com base em pesquisas anteriores (Carlotto, 2013, 2014), sabemos que isso impacta toda a trajetória profissional. Primeiro, porque a formação original está associada a um processo importante de socialização que ajuda a explicar, justamente, a relação identificada na literatura entre escolas de elite e escolas de poder (Carlotto, 2014, 2018). Em segundo lugar, a formação de graduação relaciona-se fortemente com a formação de pós-graduação e, esta última, com a área de atuação universitária (Carlotto, 2013). E, por fim, porque o padrão de carreira universitária do polo profissional tradicional é substancialmente diferente do padrão do polo acadêmico-científico, com o primeiro marcado por baixo percentual de docentes em dedicação exclusiva à vida universitária e o segundo, ao contrário, com alto percentual de professores em dedicação exclusiva (Carlotto, 2014). Nesse sentido, embora a origem da nomeação possa ser diferente – em alguns casos, mais ligada à trajetória acadêmica, em outros, à trajetória acadêmica em instituições militares e, por fim, em casos minoritários, à trajetória burocrática dentro do aparelho de Estado –, o que nos interessa destacar aqui é a dimensão da formação de graduação que permite identificar um ponto de contato entre todas essas trajetórias.

Por fim, outra divisão interessante e que merece destaque é a regional. Nesse sentido, embora os dirigentes oriundos do Sudeste sejam maioria no período (oito, ou seja, 38% do total), há uma presença importante de dirigentes oriundos do Nordeste na amostra (seis, ou 29%), resultado da sua forte presença na direção do CNPq. As demais regiões, vale notar, têm peso consideravelmente menor: só um vem do Sul, ao passo que dos dirigentes que tiveram sua origem identificada, nenhum era proveniente do Norte ou do Centro-Oeste.

Os dados sobre a origem regional dos dirigentes da PCT nacional permitem esboçar uma hipótese de que entre as três principais instituições de formulação e execução da política de ciência e tecnologia do país durante a ditadura militar havia um certo “federalismo”, com o Nordeste hegemonizando o CNPq, e o Sudeste, a Finep, ficando a Capes aberta a membros oriundos de diferentes regiões, inclusive o Sul do país. Nesse “pacto federativo” não explicitado, o Norte e o Centro-Oeste estão praticamente excluídos. Essa divisão regional do trabalho de gestão da ciência e tecnologia talvez ajude a explicar por que o estado de São Paulo, tão central para a produção científica e tecnológica do país no período, tem tão pouco peso na gestão científico-tecnológica nacional durante a ditadura militar. Por outro lado, a força da Fundação de Amparo à Pesquisa do Estado de São Paulo, que, pelo seu orçamento, tem importância similar ao de uma instituição nacional, pode ajudar a explicar a exclusão dos paulistas da PCT nacional.

O presente artigo analisou o perfil social dos dirigentes da Capes, do CNPq e da Finep entre 1964 e 1985, os três órgãos principais de formulação e implementação da política de ciência e tecnologia do país no período, visto que o Ministério de Ciência e Tecnologia foi criado apenas em 1985. No mapeamento do perfil social dos dirigentes da política de ciência e tecnologia da ditadura militar, identificamos alguns padrões importantes.

Em primeiro lugar e como já era esperado, destaca-se o perfil quase exclusivamente masculino das elites dirigentes da política de ciência e tecnologia e do país durante a ditadura militar. Uma pergunta imediata que se coloca é se esse perfil mudou a partir da redemocratização, considerando também outros níveis hierárquicos desses órgãos, como conselhos e diretorias.

Outro padrão que merece realce é a forte presença de dirigentes formados e, portanto, socializados no chamado polo profissional tradicional do campo acadêmico-científico, que envolve marcadamente os cursos de medicina, direito e engenharia, além de economia e administração. Essa constatação empírica contrariou a nossa hipótese inicial de que, ao contrário do perfil dos dirigentes do ensino superior brasileiro, as elites dirigentes da política científico-tecnológica seriam, sobretudo, formadas e socializadas em cursos acadêmico-científicos, relacionados a disciplinas tradicionais como física, química e biologia. Ainda assim, a forte presença de engenheiros no universo sugere que a divisão do trabalho entre os dirigentes das políticas de ensino superior e os da política de ciência e tecnologia nesse período se deu no interior do polo profissional tradicional, ele também heterogêneo e hierarquizado.

Outra conclusão importante é que, a despeito das semelhanças, a Capes, o CNPq e a Finep também apresentam diferenças importantes. Enquanto a primeira tem um perfil em tudo mais heterogêneo (considerando em especial o curso de formação, estado e região de origem e mesmo o sexo dos dirigentes), o CNPq e a Finep tiveram, entre seus dirigentes no período, perfis mais homogêneos. Primeiro, CNPq e Finep tiveram, entre seus dirigentes, exclusivamente homens, oriundos, na sua maioria absoluta, do polo profissional tradicional do ensino superior. Além disso, enquanto o CNPq destaca-se pela presença de engenheiros, nascidos exclusivamente na região Nordeste do país, com atuação marcante na academia, a Finep pode ser considerada uma agência muito mais atrelada à burocracia estatal, com maior presença de economistas e planejadores, oriundos exclusivamente do Sudeste. Nos dois casos destaca-se um padrão mais claro e homogêneo do que a Capes, que passou por uma redefinição maior de seu papel no período.

A partir dessas conclusões, pesquisas futuras avançarão sobretudo em duas direções: (i) na reconstrução e análise das trajetórias dos dirigentes apresentados neste trabalho, com a definição mais clara e refinada de perfis e percursos por meio de novas pesquisas documentais e, quando possível, entrevistas; (ii) na identificação mais completa das elites dirigentes da política de ciência, tecnologia e inovação, com a expansão do escopo do nosso universo tanto para incorporar novos cargos e agências, com destaque para o Ministério de Ciência, Tecnologia e Inovação e as Fundações de Amparo à Pesquisa, quanto para ampliar o período analisado, partindo de 1951, ano de fundação da Capes e do CNPq, até 2016, ano de extinção do ministério.

A extinção do Ministério de Ciência, Tecnologia e Inovação e a crise de financiamento das políticas de ciência, tecnologia e inovação que se seguiu encerrou mais um ciclo da história das políticas de CTI no país. A recriação do ministério em 2023, que passou a ser ocupado pela primeira vez na história por uma mulher, abre um novo contexto, em que continua urgente realizar um balanço das iniciativas de financiamento ao desenvolvimento científico, tecnológico e tecnoprodutivo promovido no país em períodos mais recentes. Este artigo é parte desse esforço, que provavelmente mobilizará uma parte importante do campo de estudos de CTS no país.

## Data Availability

Não estão em repositório.

## References

[B1] ADLER Emanuel (1987). The power of ideology: the quest for technological autonomy in Argentina and Brazil.

[B2] ALMEIDA Ana Maria F (2008). O assalto à educação pelos economistas. Tempo Social.

[B3] ARBIX Glauco, TOLEDO Demétrio G.C. de, Turchi Lenita, De Negri Fernando, De Negri João (2013). Impactos tecnológicos das parcerias da Petrobras com universidades, centros de pesquisa e firmas brasileiras.

[B4] ÁVILA Rebeca Contrera, PORTES Écio (2009). Notas sobre a mulher contemporânea no ensino superior. Mal-Estar e Sociedade.

[B5] BOECHAT Gabriela, CARLOTTO Maria Caramez (2017). Fatores pré-universitários de socialização e a entrada na carreira de pesquisa: trajetórias femininas na UFABC.

[B6] BOURDIEU Pierre (2004). Para uma sociologia da ciência.

[B7] BOURDIEU Pierre (2002). A dominação masculina.

[B8] BOURDIEU Pierre (1984). Homo academicus.

[B9] BOURDIEU Pierre (1975). La spécificité du champ scientifique et les conditions sociales du progrès de la raison. Sociologie et Societé.

[B10] BURGOS Marcelo Baumann (1999). Ciência na periferia: a luz síncrotron brasileira.

[B11] CARLOTTO Maria Caramez (2022). Relato pessoal ou primazia da estrutura? Da história oral à história estrutural como modelo para a sociologia histórica: o caso da história institucional da USP. Tempo Social.

[B12] CARLOTTO Maria Caramez (2018). A universidade vista a certa distância: a estrutura social da USP e sua representação simbólica. Revista Política e Sociedade.

[B13] CARLOTTO Maria Caramez (2014). Universitas semper reformanda? A Universidade de São Paulo e o discurso da gestão à luz da estrutura social.

[B14] CARLOTTO Maria Caramez (2013). Veredas da mudança na ciência brasileira: discurso, institucionalização e práticas no cenário contemporâneo.

[B15] CARLOTTO Maria Caramez, GARCIA Sylvia Gemignani (2021). O Brasil como 'terreno de experimentação' da expertise gerencial: a atuação do Conselho de Reitores das Universidades Brasileiras (1966-1985). Revista Pós-Ciências Sociais.

[B16] CARLOTTO Maria Caramez, GARCIA Sylvia Gemignani (2018). Novos saberes, novas hierarquias: disputas contemporâneas em torno da profissão acadêmica. Revista Brasileira de Ciências Sociais.

[B17] CARLOTTO Maria Caramez, HITNER Verena, Ramirez René (2018). La investigación científica y tecnológica y la innovación como motores del desarollo humano, social y económico para América Latina y el Caribe.

[B18] CASTRO Claudio Moura, SOARES Gláucio (1983). Avaliando as avaliações da Capes. Revista de Administração de Empresas.

[B19] CHARLE Christophe, Heinz Flávio (2006). Por uma outra história das elites.

[B20] COSTA Maria Conceição da (2006). Ainda somos poucas: exclusão e invisibilidade na ciência. Cadernos Pagu.

[B21] DAGNINO Renato (2007). Ciência e tecnologia no Brasil: o processo decisório e a comunidade de pesquisa.

[B22] DIAS Rafael de Brito (2012). Sessenta anos de política científica e tecnológica no Brasil.

[B23] DOMINGOS Manoel (2004). Trajetória do CNPq. Acervo.

[B24] ELZINGA Aant, JAMISON Andrew, Jasanoff Sheila (2000). Handbook of science and technology studies.

[B25] ERBER Fábio, GUIMARÃES Eduardo Augusto, ARAÚJO José Tavares de (1985). A política científica e tecnológica.

[B26] FERRARI Amílcar Figueira (2002). O Fundo Nacional de Desenvolvimento Científico e Tecnológico (FNDCT) e a Financiadora de Estudos e Projetos (Finep). Revista Brasileira de Inovação.

[B27] FERREIRA Luiz Otávio (2008). Institucionalização das ciências, sistema de gênero e produção científica no Brasil (1939-1969). História, Ciências, Saúde - Manguinhos.

[B28] FERREIRA Marieta de Moraes, MOREIRA Regina da Luz (2002). Capes 50 anos: depoimentos ao CPDOC.

[B29] FORJAZ Maria Cecília (1989). Cientistas e militares no desenvolvimento do CNPq (1950-1985). BIB, Revista Brasileira de Informação Bibliográfica em Ciências Sociais.

[B30] GARCIA Sylvia Gemignani, CARLOTTO Maria Caramez (2013). Tensões e contradições do conceito de organização aplicado à universidade: o caso da criação da USP-Leste. Avaliação.

[B31] GARCIA Sylvia Gemignani, CARLOTTO Maria Caramez, Pinheiro Rômulo, Benneworth Paul, Jones Glenn (2012). Universities and regional development: a critical assessment of tensions and contradictions.

[B32] GODIN Benoit (2006). The linear model of innovation: the historical construction of an analytical framework. Science, Technology, & Human Values.

[B33] GODIN Benoit (2004). The new economy: what the concept owes to the OECD. Research Policy.

[B34] GUEDES Moema de Castro (2008). A presença feminina nos cursos universitários e pós-graduações: desconstruindo a ideia de universidade como espaço masculino. História, Ciências, Saúde - Manguinhos.

[B35] HAMILTON Wanda, FONSECA Cristina (2003). Política, atores e interesses no processo de mudança institucional: a criação do Ministério da Saúde em 1953. História, Ciências, Saúde - Manguinhos.

[B36] HEY Ana Paula (2008). Esboço de uma sociologia do campo academico: a educação superior no Brasil.

[B37] HEY Ana Paula, CATANI Afrânio, Morosoni Marília (2006). A universidade no Brasil: concepções e modelos.

[B38] LACAZ Carlos da Silva, MAZZIERI Berta Ricardo de (1995). A Faculdade de Medicina e a USP.

[B39] LIMA Ana Luce, PINTO Maria Marta (2003). Fontes para a história dos 50 anos do Ministério da Saúde. História, Ciências, Saúde - Manguinhos.

[B40] LIMA Mozart (2003). A saúde entre o Estado e a sociedade. [Entrevista concedida a Carlos Fidelis Ponte.]. História, Ciências, Saúde - Manguinhos.

[B41] LIMA Nísia Trindade, FONSECA Cristina, SANTOS Paulo Roberto (2004). Uma escola para a saúde.

[B42] LIMONGI Fernando, Miceli Sergio (1989). História das ciências sociais no Brasil.

[B43] MARTINS Ana Luiza, BARBUY Heloísa (1998). Arcadas: história da Faculdade de Direito da Universidade de São Paulo.

[B44] MICELI Sergio, Miceli Sergio (1989). História das ciências sociais no Brasil.

[B45] MOREIRA Giovanna, CARLOTTO Maria (2023). A mudança estrutural do espaço intelectual brasileiro chegou no topo do sistema científico? O perfil dos dirigentes da CAPES, CNPq e FINEP, das origens aos dias de hoje.

[B46] MOTOYAMA Shozo (2006). USP 70 anos, imagens de uma história vivida.

[B47] MOTOYAMA Shozo (2002). 50 anos do CNPq contados pelos seus presidentes.

[B48] MOTOYAMA Shozo (1999). Fapesp, uma história de política científica e tecnológica.

[B49] NAKATA Vera (2007). Escola de engenheiros e de líderes.

[B50] SCHWARTZMAN Simon (1979). Formação da comunidade científica no Brasil.

[B51] SHARIF Naubahar (2006). Emergence and development of the national innovation systems concept. Research Policy.

[B52] SHINN Terry (2002). Nouvelle production du savoir et triple hélice. Tendences du prêt-à-penser las sciences. Actes de la Recherche en Sciences Sociales.

[B53] TOLEDO Demétrio G.C. de (2020). Dependência e autonomia nas políticas externa e tecnológica do Brasil, 1951-79. Monções: Revista de Relações Internacionais da UFGD.

[B54] TOLEDO Demétrio G.C. de (2019). Aspectos históricos e conceituais da dependência tecnológica da América Latina sob o novo neocolonialismo. Oikos: Revista de Economia Política Internacional.

[B55] TOLEDO Demétrio G.C. de (2015). Catch-up, tecnologia, instituições e empresas: desenvolvimento como aquisição de competências?. BIB, Revista Brasileira de Informação Bibliográfica em Ciências Sociais.

[B56] VIOTTI Eduardo, Velho Lea, Paula Maria Souza (2008). Avaliação de políticas de ciência, tecnologia e inovação: diálogo entre experiências internacionais e brasileiras.

